# Family based behavioral treatment in adolescents suffering from obesity: evolution through adulthood

**DOI:** 10.1186/s12887-023-04497-x

**Published:** 2024-01-10

**Authors:** Catherine Chamay Weber, Claudine Gal-Duding, Albane BR Maggio

**Affiliations:** 1 Health and Movement Consultation, Division of Pediatric Specialties, Department of Pediatrics, Gynecology and Obstetrics, Geneva University Hospitals and University of Geneva, Geneva, Switzerland; 2 Adolescent Health Unit, Division of General Pediatrics, Department of Pediatrics, Gynecology and Obstetrics, Geneva University Hospitals and University of Geneva, Geneva, Switzerland

**Keywords:** Adolescents, Obesity, Treatment, Long term evolution, Transition of care

## Abstract

**Background:**

Family Based Behavioral Treatments (FBBT) are reported to have a favorable impact on the short and mid-term evolution on the body mass index (BMI) of adolescents suffering from obesity. This study investigated the long-term BMI z-score evolution, as well as variables associated with favorable or unfavorable evolution in adolescents who beneficiated from FBBT group therapy treatment for obesity.

**Methods:**

This was a prospective study including adolescents who participated in FBBT group therapy for obesity sessions (n = 131). All adolescents were invited for a study’s clinical interview 4 years after the therapy, during which their weight and height were measured, and they answered a questionnaire on some life habits. Anthropometric measurements at the time of therapy as well as socio-demographic data were retrospectively extracted from the electronic medical record.

**Results:**

Seventy-six subjects (57% of the sample) accepted to participate in the study. At the study’s clinical interview (mean 5.5 years after FBBT), 52.6% of the adolescents showed a favorable evolution of their weight status defined as a decrease (>-0.2) or stabilization (between − 0.2 and + 0.2) of their BMI z-score. 32% were engaged in a daily physical activity and 40.6% monitored their weight at least once a week or more. Only these 2 variables were associated with a favorable evolution (*p* = 0.009 and *p* = 0.001, respectively). Less than half of the sample (45.9%) have maintained a medical weight-management follow up, of which 67% had a BMI equal or more than 30.

**Conclusions:**

Long-term evolution of the BMI z-score was favorable for most of the adolescents, emphasizing the potential benefits of FBBT treatment on the long term for adolescents suffering from obesity. This study highlighted the difficulty for long-term weight management follow up in this population at risk of numerous medical comorbidities, confirming the need to improve adherence to weight management treatment at this age of transition of care. Prospective observational study registered.

**Supplementary Information:**

The online version contains supplementary material available at 10.1186/s12887-023-04497-x.

## Background

Obesity is a complex chronic disease which is challenging to manage, especially during adolescence. In addition, for many patients, being overweight at this age is a major risk factor for further weight gain through young adulthood, resulting in significant short- and long-term health consequences [1, 2]. Therefore, implementation of healthy behaviors during this critical developmental period is of crucial importance [3].

Comprehensive, behavioral lifestyle modifications involving parents, such as FBBT, have been shown to be more successful as first line treatments for children and adolescents [4–6]. However there is little evidence on effects lasting beyond 18 to 24 months, as most studies did not carry out longer follow-up [5–10]. As adolescence is a challenging period for establishing and maintain healthy behaviors as well as regular weight and health monitoring, a long-term follow up study in this population is of particular interest [11].

The aim of this study was to investigate the body mass index (BMI) z-score evolution on the long term, as well as variables associated with a favorable or unfavorable evolution in adolescents suffering from obesity who had benefited from an FBBT group therapy.

## Methods

### Study design and subjects

This is a prospective study conducted between 2016 and 2019 on adolescents suffering from obesity who participated in FBBT group therapy at the pediatric obesity consultation of the University Hospitals of Geneva. We included all those who entered the FBBT program at least 4 years before the study period inclusion. There were no exclusion criteria. Therefore, the study population consisted of 131 adolescents who participated in the program between September 2009 and June 2014. They were all contacted by phone by a member of the obesity team (research nurse/medical student) and invited for a clinical interview with the research nurse at the hospital. After informed consent was obtained, they were asked to answer a questionnaire, were weighed, and had their height measured.

**Fig. 1:**
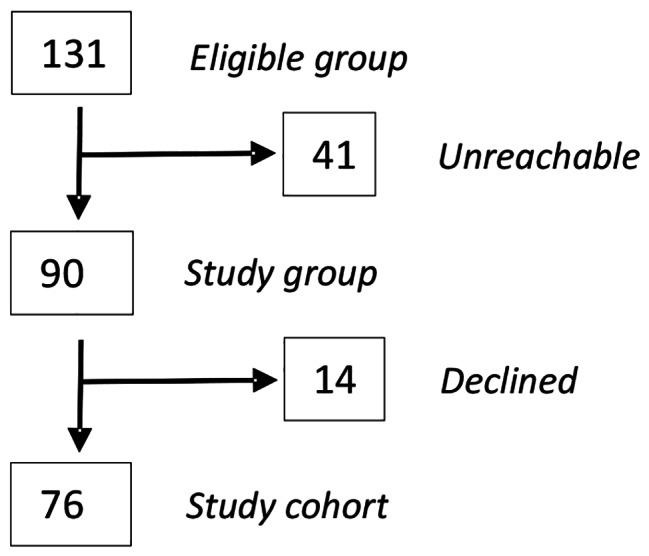
Consort flow chart

Among the 131 subjects eligible for the study, 90 (68.2%) of them were successfully reached by phone, of whom 14 (15.5%) declined and 76 (84.4%) accepted to partake in the study. This was an acceptance rate of 76/90 (84.4%) (Fig. 1).

Three study times were defined: pre FBBT (T0), and post FBBT (T1), data retrospectively extracted from the EMR of each participant and the study’s clinical interview (T2).

### Family based behavioral group therapy (FBBT)

The program consisted of 18 psycho-educative sessions for adolescents with obesity (BMI > 97 percentile), 8 for their parents and 28 sessions of physical activity over a 1-year period. The sessions were conducted by a dietician, a psychologist, and an adapted physical education teacher. The main goal of the therapy was to encourage lifestyle changes using an integrative approach including psychoeducation, stimulus control, behavioral awareness, small objectives settings, and role play. The physical activity consisted of one hour of swimming and one hour of moderate to vigorous intensity indoor exercise (ball games, cardio exercises on fitness equipment, training stations). The program for parents consisted of nutrition-related topics and systemic interventions to facilitate family functioning. At the end of the program, a regular follow-up by a health professional (physician, nurse, or dietician) was proposed, in order to maintain motivation and monitoring of weight and health. A detailed description of the group program has been published elsewhere [12].

### Questionnaire

The questionnaire was self-developed by the obesity research team as no standardized questionnaire could meet our aims. It was a structured questionnaire with multiple choice questions or dichotomous. It consisted of 13 questions on various topics such as diet and physical activity changes during the group, the maintenance of those changes, participant weight monitoring habits, and their medical weight-management follow up at the time of the clinical interview (T2) (questionnaire in appendix).

### Anthropometrics

Weight and height were measured pre FBBT (T0) and at the end of the therapy (T1) as part as the routine monitoring during the program, as well as for the study’s clinical interview (T2). Body weight (kg) and height (cm) were measured in underwear and reported in the electronic medical record (EMR). BMI was calculated as weight/height squared (kg/m^2^), and z-scores were derived using the World Health Organization references [13]. Overweight was defined as BMI z-score between 1 and 1.99 SD and obesity above 2 SD.

To define the weight status evolution, the BMI z-score changes were categorized into three groups: (1) favorable evolution if BMI z-score decreased by more than 0.2, reported to be a clinically significant response in studies; (2) stabilization if the BMI z-score remained between − 0.2 and + 0.2, considered as a favorable first step; (3) unfavorable evolution if BMI z- score increased by more than 0.2 [14, 15].

### Sociodemographic data

Socio demographic data and length of the medical weight-management follow up at the obesity consultation were retrospectively extracted from the EMR.

The Cantonal Ethics Committee approved the study, and a written informed consent was obtained from all subjects, who were all older than 16 years at the time of the study inclusion (at the study’s clinical interview, T2).

### Statistical analysis

Statistical analyses were performed using the SPSS software 25.0 (Chicago, IL). Descriptive analyses were performed using frequency distributions for the qualitative variables and mean and standard deviation (SD) for the quantitative ones. We used independent Student’s t-test, Chi-2, paired t-test and bivariate Pearson correlation when appropriate. Differences were considered significant if *p* < 0.05.

## Results

Characteristics of the 76 participants are detailed in Table 1. Mean time between T0 and T2 was of 5.5 years (± 1.03). The mean duration of the medical follow-up at the obesity consultation after the program (T1 - T2) was of 2.73 years (SD ± 0.57).

**Table 1:** Participants characteristics

Demographic	Total sample n = 76 % (n)		
**Gender**			
Girls	63 (48)		
Boys	37 (28)		
**Nationality**			
Swiss	65.8 (50)		
European	25 (19)		
Non-European countries	9.1 (7)		
**Education**			
Primary level^1^	17 (13)		
Upper secondary level^2^	60.5 (46)		
Tertiary level^3^	22.4 (17)		
**≥ 1 family member with obesity**			
First degree	53.9 (41)		
Second degree	37 (27)		
	**T0**	**T1**	**T2**
**Mean Age years (SD)**	14.3 ± 1.8	15.22 ± 1.8	20 ± 1.9
**Mean BMI (SD)**	30.85 ± 4.4	30.18 ± 4.6	33.13 ± 6.2
**BMI z-score (SD)**	2.67 ± 0.64	2.43 ± 0.69	2.50 ± 1.04

^1^Compulsory School

^2^Vocational education and training/general education

^3^Higher vocational education/university

Abbreviations: BMI: body mass index; T0: pre FBBT; T1: post FBBT (one year); T2: study’s clinical interview; zs: z-score

### BMI z-score evolution

**Fig. 2:**
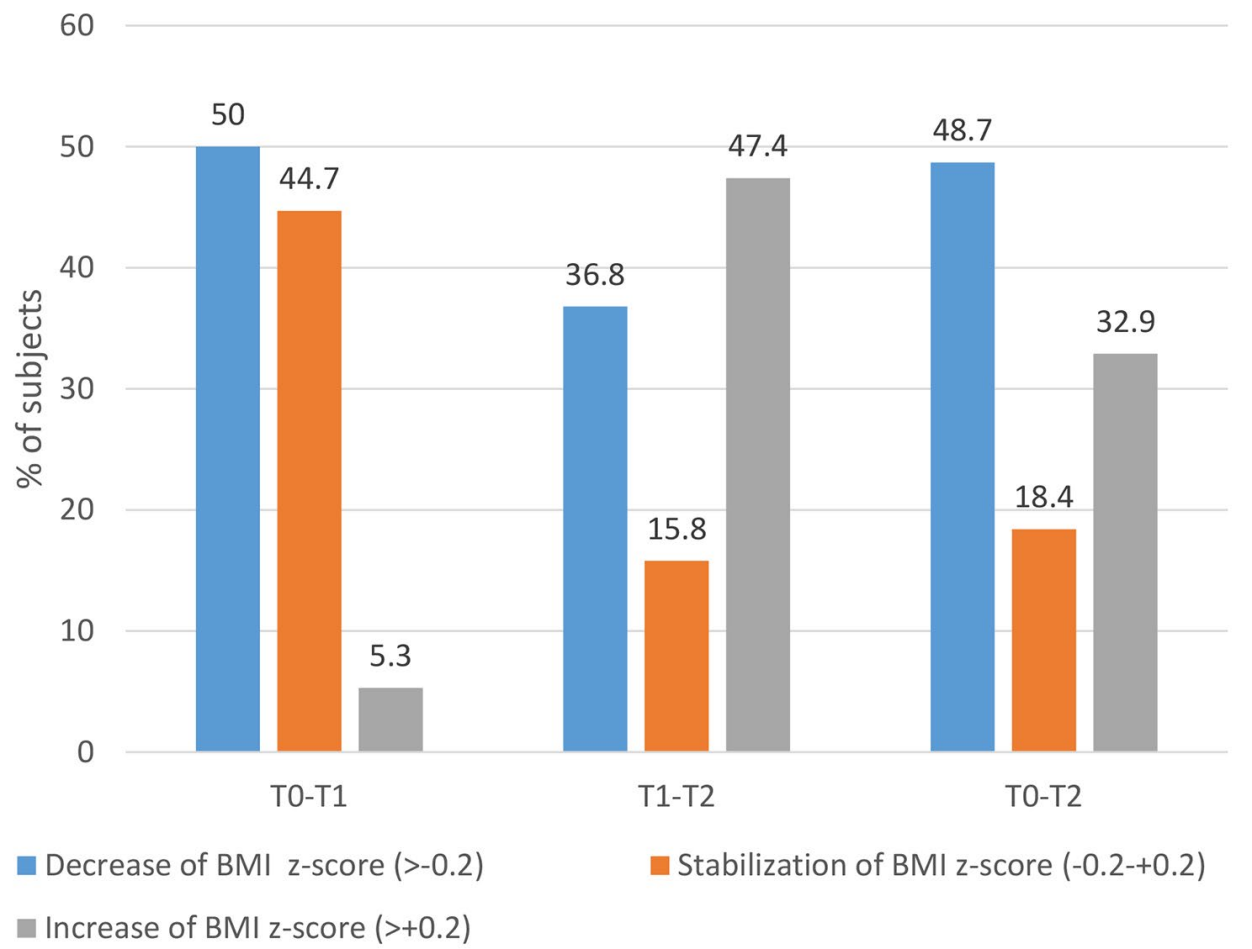
Evolution at different time points of the three BMI z score evolution. (T0: Pre FBBT; T1: Post FBBT; T2: Study’s clinical interview)

**Fig. 3:**
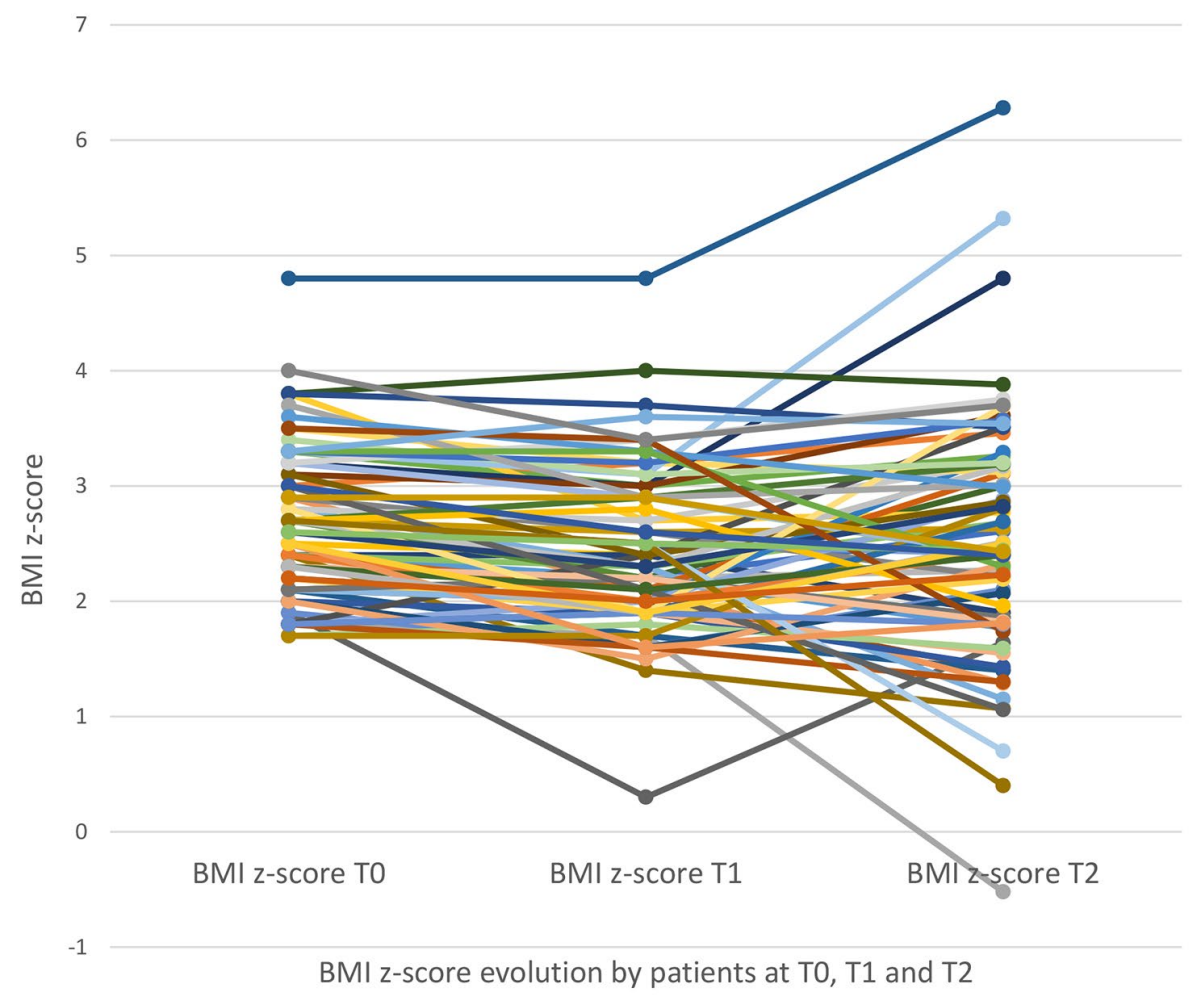
BMI z-score for each patient at pre (T0), post (T1) FBBT and study’s clinical interview (T2)

During the therapy (T0-T1), half (50%) of the adolescents decreased their BMI z-score of more than 0.2 and 44.7% stabilized it. Between the end of the therapy and study’s clinical interview (T1-T2), 52.6% had a favorable evolution with a decrease or stabilization of their BMI z-score. Few adolescents (5.3%) increased their BMI z-score of more than 0.2 points during the program (T0–T1), but this percentage increased substantially after the program (T1–T2: 47.4%). Ultimately, a little less than half of the adolescents decreased their BMI-z score and a fifth stabilized it during the study period. Only a third of them substantially increased their BMI-z score (Fig. 2). Evolution of the BMI z-score for each patient at pre FBBT (T0), post FBBT (T1) and the study’s clinical interview (T2) is presented in Fig. 3.

### Lifestyles habits evolution

60% of the subjects (n = 47) reported to be regularly physically active. 32.2% stated to perform physical activity every day, 45.8% once or twice a week, 3.4% 2–3 times per month and only 18.6% of the subjects reported to be completely inactive.

Among diet changes, 39% reported having maintained some diet changes since the therapy, which were mostly a reduction in consumption of sweet beverages (51.4) and snacking (56.9%).

### Medical follow up and weight monitoring

At the study ‘s clinical interview (T2), less than half of participants (45.9%) had an ongoing medical weight-management follow up. Among those who didn’t maintain one, 67.5% were still in obesity with a BMI equal or greater than 30. The reasons given for drop-out were: 1) a lack of motivation (19.7%), 2) good satisfaction with the treatment’s result (11.3%), 3) not having been contacted for a weight monitoring (9.9%), 4) not having been satisfied with the treatment (8.6%), 5) embarrassment for not having changed lifestyle habits (8.5%), 6) a lack of time (8.5%), 7) related to the costs (5.6%). None of them reported an interruption because of the lack of knowledge of whom to contact. About half of them (44.8%) would have been interested in restarting a follow-up for their weight at T2. Among those who did not want to restart a follow-up, 75% had a BMI greater than 30. Among those who wanted to restart a follow up, 57.7 had a BMI of more than 30.

Regarding self-weight monitoring, 45% did not know their weight at T2. Among them, 30.5% never weighted themselves, especially those who were the most overweight, which concerned 75.8% of the subjects with a BMI above 30. 29% weighed themselves monthly or less. 72% of them had a BMI greater than of 30. 41% reported a frequent weighing either weekly (27%) or daily (13.6%).

### Association between socio-demographic variables, health habits, weight monitoring and favorable or unfavorable evolution at the long-term visit

To compare the characteristics of the subjects with favorable or unfavorable evolution, we regrouped the 2 categories, decrease and a stabilization of the BMI z-score between post FBBT (T1) and the study’s clinical interview (T2), in a favorable evolution group, and the one who increased their BMI z-score, in an unfavorable evolution group. Table 2 presents the comparison of different variables between the 2 groups. The associated variables with a favorable evolution were to be engaged in a physical activity every day (*p* = 0.009) and to monitor their weight once a week or more (*p* = 0.001). Of note, a trend to statistical significance for regular weight monitoring (*p* = 0.080), physical activity once a week or more (*p* = 0.054) and increase in consumption of vegetable and fruits (*p* = 0.083) was observed.

**Table 2:** Comparison between subjects with favorable or unfavorable evolution between the end of the FBBT and the study’s clinical interview

	Favorable evolution n = 40 (52.6%))	Unfavorable evolution n = 36 (47.4%)	*p*
**Subjects’ characteristics**	**Mean (SD)**	**Mean (SD)**	
Age at T0, years	14.0 (1.8)	14.7 (1.7)	0.110
BMI at T0, kg/m2	30.7 (4.6)	31 (4.2)	0.731
BMI z-score at T0	2.7 (0.6)	2.6 (0.7)	0.691
Follow up duration (years)	2.25 (1.58)	2.08 (1.5)	0.655
Time between T0 and T2 (years)	5.7 (1.1)	5.4 (0.92)	0.232
	**n (%)**	**n (%)**	
Gender			
- Male	16 (40.0)	12 (33.3)	0.547
- Female	24 (60.0)	24 (66.7)	
Obesity 1er degree			
- Yes	33 (82.5)	25 (69.4)	0.181
- No	7 (17.5)	11 (30.6)	
Treatment response at T1			
- Favorable	38 (95.0)	34 (94.4)	0.914
- Unfavorable	2 (5.0)	2 (5.6)	
**Lifestyle habits**	**n (%)**	**n (%)**	
Diet changes			
- Still on going	14 (40.0)	14 (38.9)	0.924
- No more	21 (60.0)	22 (61.1)	
- missing value n = 2			
Physical activity once a week or more			
- Yes	28 (87.5)	18 (66.7)	0.054
- No	4 (12.5)	9 (33.3)	
- missing value n = 17			
Physical activity everyday			
- Yes	15 (46.9)	4 (14.8)	**0.009**
- No	17 (53.1)	23 (85.2)	
- missing value n = 17			
Self-Weight monitoring			
- Once a week or more	19 (61.3)	5 (17.9)	**0.001**
- Less than once a month or never	12 (38.7)	23 (82.1)	
- Missing value n = 17			

Abbreviations: BMI: body mass index; T1: post FBBT; T2: Study’s clinical interview

## Discussion

This study showed a favorable long-term evolution of the BMI z-score for most of the young adults suffering from obesity who participated in a FBBT during their adolescence. These results might suggest the long term benefits of FBBT at this age, which is recognized to be effective in children, but with less evidence in adolescents [9]. However, effectiveness of the treatment can only be assumed, as there is no control group. Nevertheless, earlier findings showed that adolescents suffering from obesity are at risk for weight gain in adulthood with little chance of spontaneous favorable resolution [2, 16].

This study has identified two behaviors associated with a favorable evolution on the long term: to have a daily physical activity and to monitor the weight regularly. Other factors such as age, gender, or other socio-demographic determinants, as well as initial BMI, treatment response at the end of the one-year program or follow-up duration after the program were not associated with a favorable or unfavorable evolution. Dietary changes were not associated with a better outcome probably because the question was too vague, without specifying the quantity or quality of these changes leaving the participants to roughly estimate them.

The impacts of regular physical activity on weight for both adolescents and adults are well studied. In our study, being active every day predicted the best evolution of BMI-z-score as opposed to once a week or less, in line with the guidelines on pediatric obesity management recommending 30 min of moderate-to-vigorous physical activity per day, 5 days per week for adolescents [7, 9, 17, 18]. However, this study did not measure the intensity of the activity which would have been interesting to evaluate if our population met the recommended guidelines.

Monitoring weight regularly was associated with a favorable evolution on the long-term. This is consistent with earlier findings identifying self-weighing as a useful weight control strategy [19–22]. However, conclusions differed on the psychological impact of this behavior. Zheng and al. reported no association between self-weighing and negative psychological outcome, while Pacanowski et al. had a more balanced conclusion, with negative psychological outcomes particularly in younger participants, and an association with the occurrence of disordered eating. Studies focusing on adolescents supported these conclusions and recommended a cautious use of regular weighing in that population that should be supported by a professional and in a particular setting (weight loss program versus normal weight adolescents) [19, 23–26]. In our study, we did not evaluate psychological impacts of self-weighing and thus cannot draw any conclusions. However, our results supported regular self-weighing as a part of a weight management strategy for young adults who were included in an FBBT when they were younger [24, 27].

Interestingly, participants with a higher BMI were the least prone to self-weighing. This may be explained by the emergence of negative emotions such as body shame and the feeling of not being able to lose weight after the weighing, reinforcing the need for these patients to have some support by health professional for their weight monitoring [17–20].

Regarding medical weight-management follow up during the transition to adulthood, this study showed that about half of our studied population has discontinued their medical follow-up, whereas most of them still had a BMI above 30. This result is concerning and shows the difficulties for long-term weight management at this age, even though they have a chronic disease leading to numerous somatic complications. In this study, a quarter of them explained their drop out by a lack of motivation or dissatisfaction with the program, which can be interpreted as a feeling of helplessness/lassitude regarding weight loss efforts. Transition of care is known to be problematic in this population [11]. It is argued by the fact that most of the time, adolescents do not stay in treatment long enough to transition, which is also the case in our study, with a mean follow up of 2.7 years. One of the reasons might be that they or their parents do not identify obesity as a health problem, but more as a behavioral issue or a lack of motivation that does not need to be supported by a health professional. Another reason might be that they have not succeeded in making lifestyle changes to lose weight, despite several years of weight management, leading to a feeling of ineffectiveness and lassitude, resulting in a demotivation for treatment both for the youth and their parents. Asking for assistance may also be viewed as difficult and embarrassing. Cost for the patient could be a reason for a drop out after 18, as the Swiss health insurance system can require substantial contributions to medical cost after majority, whereas before 18, costs are covered by the insurance. However, few of them reported this limitation in our questionnaire. Surprisingly, half of our participants would have been interested in restarting a follow-up if offered at the long-term visit. These results indicated that health professionals should be more proactive with these patients, offering them an appointment at regular intervals, even if they are often considered unnecessary by healthcare providers and/or parents. Ensuring that the patient has made the transition to adult care could also be a way of restarting an interrupted follow-up. Clinical guidance on transition care in adolescents with obesity should be developed as proposed by Shrewsbury and al in order to be more efficient with those patients [11].

This study has some limitations. First, the sample was small, with a high number of adolescents who did the program but didn’t participate in the study, even if the main reason for not participating was that they were unreachable. Therefore, a recruitment bias cannot be excluded. Those not included were analyzed at baseline (T0) as well as their evolution at the end of their participation in the group therapy. At baseline, the mean age was similar to the study group (14.1 ± 1.3 vs. 14.3 ± 1.8 years). They were a somewhat more overweighted at baseline (BMI 31.61 ± 4.8 vs.30.85 ± 4.4; BMI-z score 2.67 ± 0.64 vs. 2.43 ± 0.69). The evolution of the BMI z-score between T0 and T1 was favorable in 92.8% of them versus 94.7% in the study group. Only few adolescents increased their BMI z-score as in the study group (7.3% vs. 5.3). The main difference was the gender distribution, with substantially more boys (60% vs. 34%) in the group who didn’t participate in the study.

Among those who could be reached, 84% did participate, suggesting that the study was well perceived. Furthermore, the adolescents did not know at the end of the program that they would be contacted for a long-term clinical evaluation, and thus we were not able to refer to drop-out rates per se. Finally, drop-out rate is a common problem in adolescent obesity studies, which probably explains the few studies on the long-term evolution (more than 2 years) in this population emphasizing our results.

Another limitation is the use of BMI z-score, reported to be a poor indicator of adiposity among youths in some recent publications, particularly those with severe obesity [9]. However, no standard definition of weight loss success exists for the pediatric populations [7, 9].

Finally, the changes in the intensity of physical activity and diet habits have not been fully addressed in this study, preventing us from making any robust conclusions. However, we would have probably lost a certain number of participants if they had been asked to wear a device to measure their amount of physical activity and intensity. For diet habits, it is known that even with food recall questionnaire, this issue is difficult to address.

Despite these limitations, this study is one of the few on the long-term evolution of adolescents suffering from obesity.

## Conclusion

FBBT might have a long-term benefit on BMI z-score, especially for those who weekly monitor their weight and are engaged in a daily physical activity. This study highlighted the frequent discontinuation of follow up and weight monitoring at this time of transition of care, particularly for those at risk of medical complications. In view of the numerous medical problems associated with obesity, health professionals must be more proactive in identifying patients with obesity dropping out during adolescence and invited them to discuss transition of care. Future studies on treatment adherence and transition of care in adolescents with obesity are necessary.

### Electronic supplementary material

Below is the link to the electronic supplementary material.

Supplementary Material 1

## Data Availability

The datasets used and/or analyzed during the current study are available from the corresponding author on request.
