# Supplementary material for: Family based behavioral treatment in adolescents suffering from obesity: evolution through adulthood
Source: BMC Pediatr. 2024 Jan 10;24:33. doi: 10.1186/s12887-023-04497-x (PMC10777574; doi:10.1186/s12887-023-04497-x)
Supplement: Supplementary file 1 — Supplementary Material 1 [file 12887_2023_4497_MOESM1_ESM.docx]

**Questionnaire for adolescents who have taken part in the FBBT group program**

Study on the evolution of adolescents suffering from obesity and treated in a specialized consultation (Protocol N° 10-045)

This research data will be treated confidentially. Your contact details will not appear.

Dear young

You took part in a group program a few years ago, and we'd like to know about your experience and current situation.

If you agree, please take a few minutes to answer the following questions, circling the answer that best describes you.

**1) In general, do you think the group program has helped you?**

*Circle one answer only*

a) A lot

b) Moderately

c) A little

d) Not at all

**2) Did the group help you change your lifestyle habits ?** *Circle one answer only*

a) A lot

b) Moderately

c) A little

d) Not at all

**3) Did the group help you change your eating habits ?** *Circle one answer only*

a) A lot

b) Moderately

c) A little

d) Not at all

**4) Which diet habits have you been able to implement?** *Several answers possible*

a) Eat breakfast more regularly

b) Cut down on sugary drinks

c) Eat vegetables/fruit more often

d) Think about balancing my meals better

e) Reduce snacking

**5) Did the group help you to engage in regular physical activity?** *Circle one answer only*

a) A lot

b) Moderately

c) A little

d) Not at all

**If yes, how many times a week :** *Circle one answer only*

a) <1x/per week

b) 1x/per week

c) 2x/per week

d) 3x/per week

e) >3x/per week

f) Every day

**6) In the event of changes (dietary or sporting) following the group, how long did you maintain them?**

*Circle one answer only*

**a) Dietary changes :**

a) ≤6 months post-group

b) ≤1 year after the group

c) ≤ 2 years after the group

d) Still ongoing

**b) Physical activity changes**

a) ≤6 months post-group

b) ≤1 year post-group

c) ≤2 years post-group

d) Still in progress

**7) Do you currently do regular physical activity?**

Yes □ No □

**How often?** *Circle one answer only*

a) Every day

b) 1-2x per week

c) 2-3x per month

d) Never

**9) Do you currently weigh yourself regularly?** *Circle one answer only*

a) Every day

b) 1x per week

c) ≥per month

d) Never

**10)** **Has your weight changed since the** **group?** *Circle one answer only*

a) I never weigh myself, so I don't know

b) I've remained stable

c) I've lost weight

d) I've gained weight

**11) Do you know your current weight and height?**

Weight in kg: Height in cm :

**12) Do you currently see a medical professional to help you monitor your weight?**

Yes □ No □

If yes, by whom?

*Several possible choices*

a) A doctor

b) A nurse

c) A dietician/nutritionist

d) Sports coach

e) Other:

**13) If you haven't maintained a medical follow up, what were the reasons?**

*Several possible choices*

a) I was satisfied with the results and no longer required follow-up for my weight

b) I was not satisfied with the care offered and did not see the need to continue a

medical follow-up for my weight

c) Lack of motivation

d) I'm embarrassed because I can't achieve or maintain the weight loss changes

e) I've never been contacted for follow-up, and I haven't taken any particular action to seek any

f) I don't know/my parents don't know who to contact

g) I don't have the time

h) Financial concerns

i) My parents didn't want me to continue with follow-up for my weight

**14) If you don't have a medical follow-up for your weight at the moment, would you be interested in restarting one?**

Yes □ No □
